# Improving the diversity of captured full-length isoforms using a normalized single-molecule RNA-sequencing method

**DOI:** 10.1038/s42003-020-01125-7

**Published:** 2020-07-30

**Authors:** Yueming Hu, Xing-Sheng Shu, Jiaxian Yu, Ming-an Sun, Zewei Chen, Xianming Liu, Qiongfang Fang, Wei Zhang, Xinjie Hui, Ying Ying, Li Fu, Desheng Lu, Rakesh Kumar, Yejun Wang

**Affiliations:** 1 School of Basic Medicine, Shenzhen University Health Science Center, Shenzhen, 518060 China; 2 College of Veterinary Medicine, Yangzhou University, Yangzhou, Jiangsu China; 3 Department of Gastrointestinal Surgery, Shenzhen People’s Hospital, The Second Clinical Medical College of Jinan University, Shenzhen, 518020 China; 4 Shenzhen GenRead Tech. Co. LTD., Shenzhen, 518132 China; 5 Rajiv Gandhi Centre for Biotechnology, Trivendrum, 695014 Kerala India; 6 Virginia Commonwealth University School of Medicine, Richmond, 23298 USA; 7 Rutgers New Jersey Medical School, Newark, 07103 USA

**Keywords:** RNA sequencing, Gastric cancer

## Abstract

Human genes form a large variety of isoforms after transcription, encoding distinct transcripts to exert different functions. Single-molecule RNA sequencing facilitates accurate identification of the isoforms by extending nucleotide read length significantly. However, the gene or isoform diversity is lowly represented by the mRNA molecules captured by single-molecule RNA sequencing. Here, we show that a cDNA normalization procedure before the library preparation for PacBio RS II sequencing captures 3.2–6.0 fold more full-length high-quality isoform species for different human samples, as compared to the non-normalized capture procedure. Many lowly expressed, functionally important isoforms can be detected. In addition, normalized PacBio RNA sequencing also resolves more allele-specific haplotype transcripts. Finally, we apply the cDNA normalization based long-read RNA sequencing method to profile the transcriptome of human gastric signet-ring cell carcinomas, identify new cancer-specific transcriptome signatures, and thus, bring out the utility of the improved protocols in gene expression studies.

## Abstract (web summary)

Hu et al. combine cDNA normalization before library preparation with a software tool to increase the capture of RNA isoform species in single-molecule RNA sequencing. They demonstrate that this approach can detect previously unknown transcripts in gastric signet-ring cell carcinomas that are not present in non-malignant tissue.

## Introduction

Transcript identification is the primary, major objective in most of the RNA sequencing (RNA-Seq)-based transcriptome studies. Most human genes have multiple isoforms, expression of many of which are tissue-, developmental stage-, or stimulus-specific, and hence, resulting functions^1–4^. In general, the length of second-generation sequencing (SGS) reads, e.g., those from Illumina, is ~100–250 bp, much smaller than that of most human transcripts (~2500 bp)^5^. The diploid nature of mammals further complicates the accurate assembly of the short reads into long unique transcripts. The emergence and development of single-molecule sequencing (SMS) technology, e.g., PacBio RS II and Oxford Nanopore, provides solutions to the SGS short-read problem. Typically, the single SMS reads (~10 kb or longer) cover the full length of a transcript, leading to the feasibility of detecting long transcripts without read assembly step^6–10^ as well as allele-specific expression of isoforms^11^. However, the SMS method also has its own limitations. For example, the output of SMS is much lower than SGS, whereas the cost is much higher, and the longer size of individual reads further decreases the number of isoform clusters captured. The preference of shorter transcripts (~1.5 kb) and the super high expression of a few house-keeping genes worsen the situation, and the eventual number of meaningful isoforms captured could be very limited^6,11,12^. Although the output of SMS has been increased, and new library preparation strategies (e.g., size selection) were thought to improve the isoform sequencing, the problem remains.

In early years of SGS applications in transcriptome studies, the problem of high cost or low output started to surface, limiting the capture of mRNA species. Duplex-specific nuclease (DSN)-based cDNA normalization was developed, which effectively subtracted the high-expressed genes while enriched the moderately or lowly expressed ones^13^. Sequencing combined with cDNA normalization greatly increased the mRNA species. The normalization was most frequently applied for the cDNA library preparation of Roche 454 pyrosequencing, as the reads were longer, the output was lower and the cost was higher than other SGS platforms such as Illumina^14,15^. As Illumina predominates in the SGS market, normalization appeared unnecessary, as the output is huge. As SMS becomes popular and the sequencing output/cost turns back to be a major concern, it is meaningful to re-consider whether cDNA normalization could provide an ideal solution.

In this study, we systematically compare the effect of cDNA normalized and non-normalized SMS in the diversity of detected isoforms, capture of lowly expressed genes and isoform phasing. We also develop ASIIQT (Allele-Specific Isoform Identification and Quantification Tool), a software tool to phase and quantify the isoforms identified from the normalized PacBio RNA-Seq libraries. As an application, we use the cDNA normalization based SMS and paralleling SGS to profile the transcriptome of gastric signet-ring cell carcinomas (SRCCs), and identify a set of previously unknown transcripts which could possibly be developed as new cancer-specific transcriptome signatures.

## Results

### Overall design, DSN-based cDNA normalization, and sequencing

**Fig. 1:**
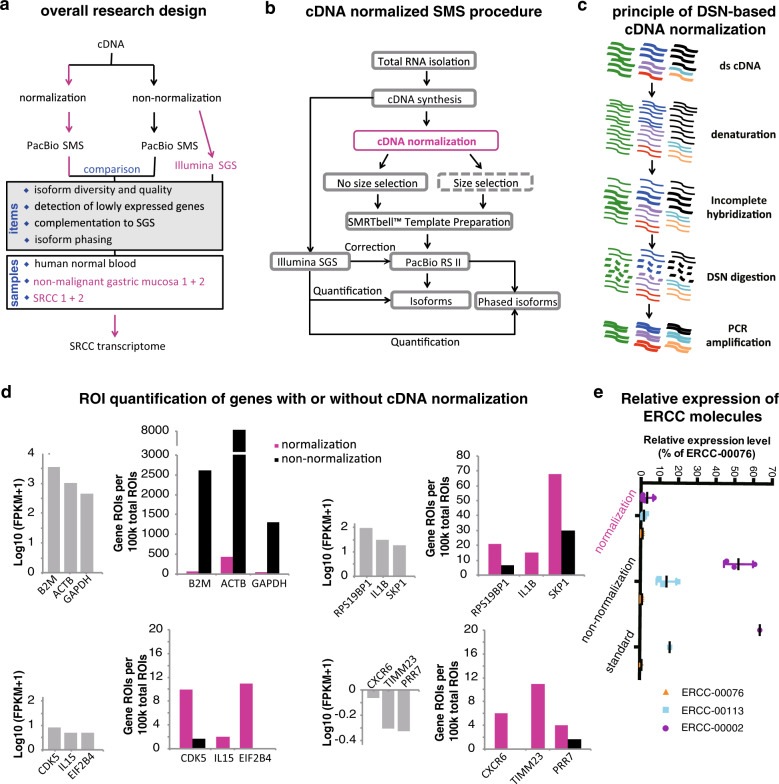
Research design and general effect of DSN-based cDNA-normalized PacBio RNA-seq. **a** Overall design of the research. Generally, cDNA generated from human peripheral blood, two SRCCs, and their adjacent non-malignant gastric mucosa samples (as listed in the white box) were normalized by DSN digestion and non-normalized, followed by PacBio RS II SMS. The effect was compared between normalization and non-normalization, mainly in respects of the four items as indicated in the gray box. Each original cDNA sample was also sequenced using Illumina SGS platforms for assisting data analysis. The SGS and normalized SMS data of SRCCs and paired samples were also used for SRCC transcriptome profiling and comparison (shown in purple). **b** General experimental and data analytic procedure of cDNA-normalized SMS. The step in the box with dash lines was optional. **c** The principle of DSN-based cDNA normalization (referring to Zhulidov et al.^13^). Basically, DSN specifically digests the incompletely hybridized double-strand (ds) cDNA molecules, which mostly represent the genes with high expression, and eventually, the transcripts of genes with uneven expression originally turn equal. **d**–**e** General effect of cDNA normalization in reducing the highly expressed genes and enriching the lowly expressed genes in human blood samples. **d** The representative genes with varied original expression (quantified by SGS, left gray bars) were quantified with the number of ROIs mapped to the corresponding genes (normalized as per 100k total ROIs) in the normalized or non-normalized SMS libraries. **e** Expression analysis of ERCC genes with real-time quantitative PCR. The graph represents the mRNA expression levels of indicated genes that were normalized to the Ct values of ERCC-00076. The experiments were performed in triplicates. “Standard” showed the actual ratio of molecular concentrations in the original reagent.

The overall study design was shown in Fig. 1a. First, we applied a DSN-based cDNA normalization procedure with an objective of increasing the diversity of isoforms detected by SMS. The effect was evaluated with multiple samples in respect of (1) isoform diversity and quality, (2) detection of lowly expressed genes, (3) complementation to short-read SGS, and (4) detection of allele-specific isoforms. Finally, we surveyed the transcriptomes of SRCCs using the proposed procedure.

For each cDNA normalization-based SMS experiment, the overall experimental and analytic procedure was shown in Fig. 1b. After RNA isolation and cDNA synthesis, cDNA aliquots were used for both SGS and cDNA-normalized SMS simultaneously. Size selection could be performed optionally after cDNA normalization. Isoforms were detected from the SMS sequences, corrected with SGS reads, and phased with the SMS and SGS data. The SGS reads were also used for quantification of the consensus and phased isoforms.

DSN was adopted to normalize the cDNA molecules (Fig. 1c). Denatured single-strand cDNA molecules were annealed incompletely and incubated with DSN, which digested the double-strand cDNA molecules formed by mostly abundant transcripts. To demonstrate the general effect of cDNA normalization, we sequenced the normalized and non-normalized human blood cDNA with PacBio RS II platform as well as the non-normalized, equal volume of initial cDNA with Illumina HiSeq 2000 platform, followed by selection of representative genes with high, moderate, and low expression levels quantified by SGS results, and compared the relative expression of these genes quantified by PacBio SMS reads (Fig. 1d). We noticed that for the highly expressed genes, e.g., *B2M*, *ACTB,* and *GAPDH*, the reads of interest (ROIs) of normalized PacBio SMS libraries were much fewer than those of non-normalized libraries. For the moderately (e.g., *RPS19BP1*, *IL1B,* and *SKP1*) or lowly expressed genes (e.g., *CDK5*, *IL15*, *EIF2B4*, *CXCR6*, *TIMM23,* and *PRR7*), however, much more ROIs were detected in normalized libraries (Fig. 1d). In independent blood RNA samples, we also added External RNA Controls Consortium (ERCC) Spike-In mixes, and then examined the relative expression of three ERCCs, i.e., ERCC-00002, ERCC-00113, and ERCC-00076, with the molecule number ratio in the original standard mixes of 64:16:1. In the non-normalized cDNA product, the relative expression of the ERCC-00002, ERCC-00113, and ERCC-00076 was 52:14:1, whereas the ratio became 3.3:1.7:1 in the normalized product (Fig. 1e). Taken together, the results confirmed that cDNA normalization could reduce transcripts with higher expression more extensively and equalized the transcripts with varied expression.

To make a comprehensive evaluation of the effect of cDNA normalization, we also tested the methods in two pairs of SRCCs and their adjacent non-malignant tissues. The statistics on the sequencing data were shown in Supplementary Data 1.

### cDNA normalization increases isoform diversity

**Fig. 2:**
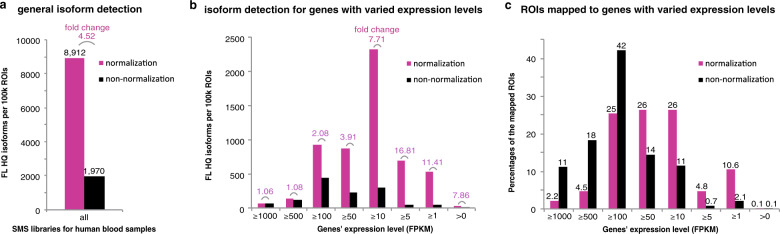
Effect comparison between cDNA-normalized and non-normalized SMS in diversity of captured isoforms. **a** Overall difference in the diversity of captured full-length high-quality isoforms (normalized as per 100k total ROIs). Fold change of the isoform number was indicated in purple. **b** Difference in the diversity of captured isoforms with varied gene expression levels. **c** Occupancy percentages of ROIs for the captured isoforms with varied gene expression. The percentages were indicated on the top of each bar. In **b**, **c** The isoform represented genes were binned according to their expression as measured by FPKM of SGS data. The SGS, normalized, and non-normalized SMS data of human peripheral blood samples were used for the analysis.

Generally, normalized SMS libraries detected more full-length high-quality isoforms than non-normalized libraries, with 8912 vs 1970 per 100k ROIs for human blood samples (Supplementary Data 1; Fig. 2a). Different biological samples demonstrated the similar advantage of cDNA normalization in detection of isoform species, with 4.21–6.99-folds of increase in general (Supplementary Data 1; Supplementary Fig. 1). We also investigated the expression distribution of detected genes quantified by SGS reads. For genes with no fewer than 500 fragments per kilobase per million reads (FPKM), the isoform detection rates, defined as the number of isoforms detected per 100k ROIs, were nearly identical between the normalized and non-normalized libraries (Fig. 2b; Supplementary Fig. 1). The advantage of isoform detection of normalization versus non-normalization, represented by the fold change of isoform detection rates, turned more obvious as the gene expression decreased. For genes with moderate expression, 10–50 FPKM for example, normalized human blood libraries could detected 7.71-fold of isoform species compared with non-normalized libraries, whereas the advantage became most typical for lowly expressed genes with <10 FPKM (Fig. 2b). The trend was identified consistently in other samples (Supplementary Fig. 1).

We also mapped the ROIs to the isoforms. Consistent with the previous results showing a large number of ROIs for a few exemplar abundant genes in non-normalized libraries (Fig. 1d), we found that, in non-normalized human blood libraries, >29% (11% + 18%) of the mapped ROIs corresponded to the few genes with highest expression of >500 FPKM, and the proportion of ROIs for genes with >100 FPKM reached 71% (Fig. 2c). In contrast, in normalized libraries, the corresponding ROIs only covered 6.7% and 32%, respectively (Fig. 2c). The malignant and non-malignant gastric samples demonstrated the same patterns (Supplementary Fig. 2). Taken together, the results supported the hypothesis that DSN-based cDNA normalization could preferably reduce the abundant transcripts and increase the overall isoform diversity.

### cDNA normalization improves isoform quality

**Fig. 3:**
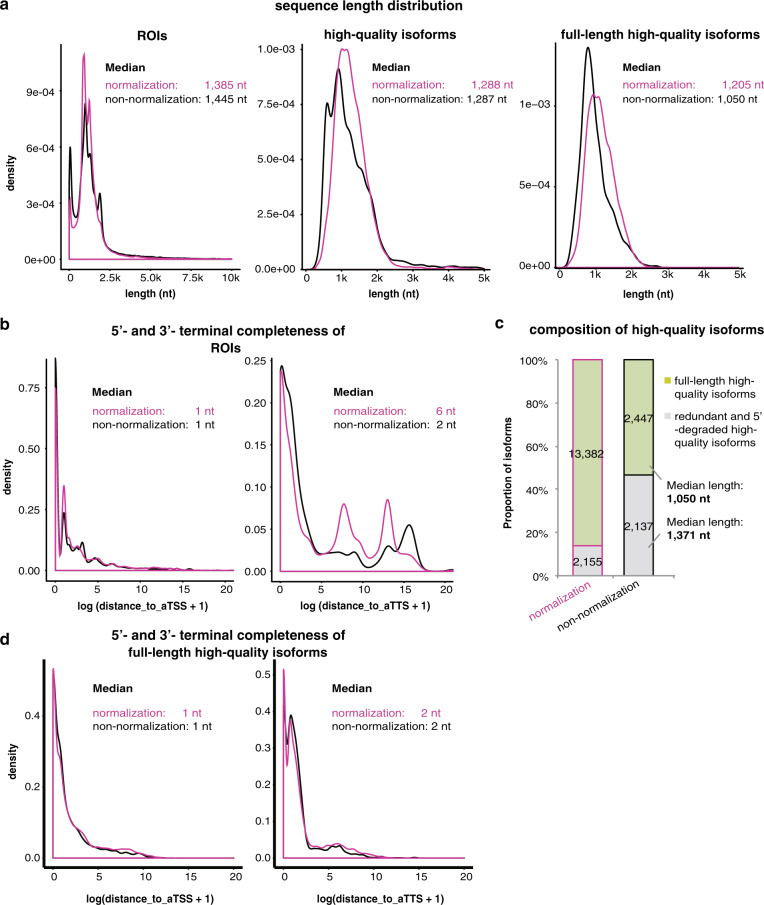
Effect comparison between cDNA-normalized and non-normalized SMS in completeness of captured isoforms. **a** Length distribution of ROIs, high-quality isoforms and full-length high-quality isoforms. The distribution of distance to annotated TSSs (aTSS, left) and annotated TTSs (aTTS, right) was shown in **b**, **d** for ROIs and full-length high-quality isoforms, respectively. In **a**, **b**, **d** the normalized and non-normalized libraries were shown in magenta and black, respectively. **c** The composition of high-quality isoforms. The high-quality isoforms retained as full-length high-quality ones were shown in green, whereas the filtered-away ones because of redundancy or 5′-degradation were shown in gray. Data of human peripheral blood samples were used for the analysis.

The quality of reads and isoforms was also compared between the libraries, in terms of length, completeness, and splice junction calling of sequenced transcripts. A similar length distribution pattern was disclosed for ROIs, but the non-normalized libraries showed a larger median (1455 nt vs 1385 nt) (Fig. 3a). To understand possible factors explaining the observed shortness of normalized ROIs, the ROIs were mapped to the full-length transcripts with annotated transcript start sites (aTSSs) and transcript termination sites (aTTSs) curated in GENCODE database. The distance between ROIs’ starting nucleotides and aTSSs was not apparently different between the libraries (Fig. 3b). However, we found a longer nucleotide distance in the normalized libraries between the ROIs’ termination sites and aTTSs as compared with the non-normalized libraries (median: 6nt vs 2nt; Fig. 3b), suggesting a possible 3′-breakdown of the transcripts during normalization.

ROIs were clustered into high-quality isoform clusters, filtered by sequence quality and 3′-end completeness (Methods). The high-quality isoforms showed similar length between the libraries (Fig. 3a, middle). High-quality isoforms were further collapsed with ToFU to merge the molecules with a unique origin and to further filter the 5′-degraded transcripts, generating the full-length high-quality isoforms (Methods). A larger proportion of high-quality isoforms were always filtered out from non-normalized libraries (Fig. 3c; Supplementary Fig. 3), indicating the relatively higher sequence redundancy and possibly more 5′-degraded isoforms being collapsed. The filtered-away high-quality isoforms often showed larger average length than the full-length high-quality isoforms in non-normalized libraries (Fig. 3c). The eventual full-length high-quality isoforms from normalized libraries were generally longer than those from non-normalized libraries (Fig. 3a; Supplementary Fig. 3). The 5′- and 3′-completeness of full-length high-quality isoforms appeared no apparent difference between the two types of libraries (Fig. 3d; Supplementary Fig. 3). Besides the transcript borders, the full-length high-quality isoforms in normalized libraries also disclosed much more splice junctions and slightly higher accuracy for the called junctions, as demonstrated by the better consistency with validated ones, and by the larger proportion of canonical ones (Supplementary Table 1).

Taken together, the results demonstrated a slightly more-extensive transcript 3′-breakdown in normalized SMS libraries and also shorter ROIs. However, the full-length high-quality isoforms showed longer sequences and better quality for normalized libraries. Taking into account the end use of full-length isoforms and the dramatically increased isoform diversity, cDNA normalization showed an apparent advantage.

### cDNA normalization captures more and lowly expressed genes

**Fig. 4:**
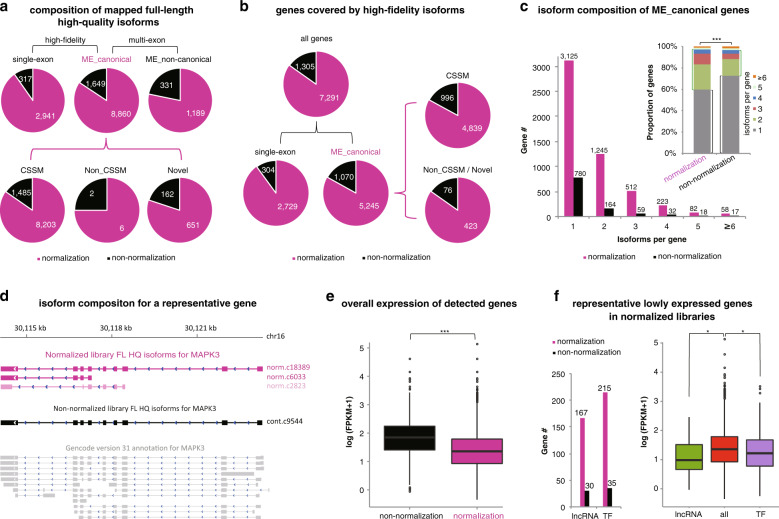
Effect comparison between cDNA-normalized and non-normalized SMS in diversity of captured genes, and in detection of moderately or lowly expressed genes. **a** Comparison of full-length high-quality isoforms classified into different subgroups. In normalized or non-normalized libraries, the full-length high-quality isoforms were classified into single-exon and multi-exon groups. The multi-exon isoforms were further subdivided into “ME_canonical” subgroup with all canonical splice sites, and “ME_non-canonical” subgroup with non-canonical splice sites. The single-exon and “ME_canonical” isoforms were together considered as high-fidelity ones. “ME_canonical” isoforms were composed by consensus split-mapped molecules (CSSMs), Non_CSSMs, and novel isoforms. **b** Comparison of genes covered by high-fidelity isoforms. **c** Comparison of isoform distribution for “ME_canonical” genes. Genes were binned into three groups based on the composed isoform number (1, 2–4, ≥5) and Chi-square test was performed. **d** Gene models of the full-length high-quality isoforms detected for an example gene, MAPK3. A non-canonical isoform from normalized libraries, “norm.c2823”, was also shown, in transparency. **e** Expression comparison for the genes detected in non-normalized vs normalized libraries. Mann–Whitney *U* test was performed. **f** Comparison of representative gene clusters with important function and low expression between normalized and non-normalized libraries. lncRNA, long non-coding RNA; TF, transcription factor. The number of genes for each cluster was indicated (left). Expression comparison was performed between lncRNA/TF genes and all genes detected in normalized libraries (right). Mann–Whitney *U* tests were performed. Data of human peripheral blood samples were used for the analysis. Gene quantification was based on SGS results. For all the statistical tests, *p < 0.05; **p < 0.01; ***p < 0.005.

The full-length high-quality isoforms were classified into single-exon and multi-exon isoforms. The multi-exon isoforms were further divided into ME_canonical and ME_non-canonical isoforms, which refer to the ones with all canonical splice junctions and the others, respectively. ME_canonical isoforms were subdivided into consensus split-mapped molecules (CSMMs) for which all introns respect the splice site consensus annotated in GENCODE, Non_CSSMs, and transcripts for novel genes. The normalized human blood libraries detected much more ME_canonical, ME_non-canonical, and single-exon isoforms than non-normalized libraries (Fig. 4a). The ME_canonical and single-exon isoforms, collectively considered as high-fidelity ones, covered 7291 and 1305 genes in normalized and non-normalized libraries, respectively (Fig. 4b). The Non_CSSM or novel ME_canonical isoforms represented 423 new genes in normalized libraries and only 76 in non-normalized libraries (Fig. 4b). A more comprehensive survey was performed to the isoform distribution for each gene (Fig. 4c). Despite the overall similar distribution patterns for both types of libraries with the largest proportion of genes containing only one isoform, decreased percentages of genes for more isoforms, and most genes with less than six isoforms per gene, normalized libraries showed smaller percentages of genes with 1 and ≥5 isoforms per gene, and larger percentages for the rest, than non-normalized libraries (Chi-square test, *p* = 2.983e-11) (Fig. 4c).

To illustrate the utility of isoform detection procedure presented here, we used Mitogen-activated protein kinase 3 (*MAPK3*) as an example and demonstrated that, an increased number of isoforms were identified from normalized than non-normalized libraries (3 vs 1; Fig. 4d). Besides, we recognized a novel, previously un-annotated high-fidelity isoform only from normalized libraries, i.e., “norm.c6033” (Fig. 4d). There was also another novel full-length high-quality isoform specifically detected from normalized libraries (“norm.c6033”). However, it was classified as ME_non-canonical (Fig. 4d).

By quantifying with SGS reads, we found that genes detected in normalized SMS libraries showed significantly lower expression than those captured in non-normalized libraries (Fig. 4e; Mann–Whitney *U* test, *p* < 2.2e-16). More functionally important genes, such as lncRNAs and transcription factors, were captured in normalized libraries (167 vs 30 for lncRNAs, 215 vs 35 for transcription factors; Fig. 4f). The genes encoding lncRNAs and transcription factors also showed significantly lower expression as compared with the rest in normalized libraries (Mann–Whitney *U* tests, *p* = 0.002 for lncRNAs and 0.01 for transcription factors) (Fig. 4f), suggesting that the noted detection of lower expression transcripts in the normalized sets might be independent of global expression of transcripts.

Similarly, the normalized SMS libraries for gastric cancers and non-malignant controls detected an increased number of genes and isoform species with lower expression and important biological activity as compared to non-normalized SMS libraries (e.g., transcription regulation) (Supplementary Fig. 4).

### cDNA normalization-based SMS complements SGS methodology

**Fig. 5:**
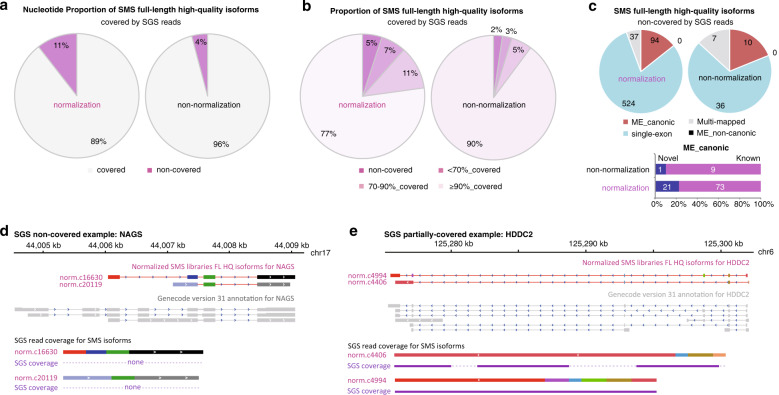
Effect comparison between cDNA-normalized and non-normalized SMS in complementation to SGS. **a** The proportion of nucleotides of SMS full-length high-quality isoforms covered or non-covered by SGS reads. **b** The proportion of SMS full-length high-quality isoforms fully-, partially-, or non- covered by SGS reads. The isoforms were classified into four groups according to the percentages of full length being covered by SGS reads. **c** Composition of the SGS non-covered full-length high-quality isoforms (upper) and the “ME_canonic” subset (lower). “Multi-mapped” isoforms were those not uniquely mapped to human reference genome and therefore not included for further grouping analysis. **d** An example gene (*NAGS*) with SMS-detected isoforms that were not covered by any SGS read. **e** An example gene (*HDDC2*) with an SMS-detected isoform that was partially covered by SGS reads. In **d**, **e** For SGS-coverage demonstration, exons for each isoform detected by normalized SMS were concatenated and introns were removed. Coverage by SGS reads was shown in purple in parallel, and the non-coverage was shown in dash lines. Data of human peripheral blood samples were used for the analysis.

SMS full-length high-quality isoforms were accurately located to the human reference genome, to which the SGS reads were also mapped. Comparative analyses were performed to estimate the coverage of SMS isoforms by SGS reads. Generally, 11% of all nucleotides of the SMS isoforms in normalized libraries and 4% in non-normalized libraries were not covered by SGS reads (Fig. 5a). In normalized SMS libraries, 5% of the isoforms were not covered by any SGS read (Fig. 5b). Approximately 77% of the isoforms were covered by SGS reads for ≥90% of the full length, whereas ~18% of the isoforms were partially covered (<90% of the full length) (Fig. 5b). In non-normalized libraries, a larger proportion (90%) of the isoforms were covered by SGS reads for ≥90% of the full length, whereas the proportions of non-covered (2%) and partially covered (8%) isoforms were both decreased (Fig. 5b).

The non-covered SMS isoforms were further analyzed and compared between normalized and non-normalized libraries. Thirty-seven (normalized) and 17 (non-normalized) were removed for further analysis because of ambiguous mapping (Fig. 5c). None of the isoforms was found with non-canonic splice junction in both types of libraries, indicating the generally high quality of the isoforms (Fig. 5c). Single-exon isoforms represented the majority, particularly in the normalized libraries (Fig. 5c). Ninety-four and 10 isoforms from normalized and non-normalized libraries respectively, were ME_canonic isoforms, and most of them represented known genes (Fig. 5c).

To illustrate the non-coverage of SMS isoforms by any SGS read, we selected *N*-acetylglutamate synthase gene (*NAGS*) as an example (Fig. 5d), of which two new isoforms were identified only in normalized SMS libraries, and neither of them was covered by any SGS read. We also selected HD domain containing 2 (*HDDC2*) to show the SMS isoforms partially covered by SGS reads. Two isoforms were detected only from normalized libraries, and one of them (norm.c4994) was completely covered by SGS reads (Fig. 5e). For the other isoform (norm.c4406), however, only fragments could be mapped (Fig. 5e).

Taken together, the results demonstrated that, despite large percentage of coverage by high-throughput SGS reads, SMS sequences could still complement additional isoforms that were missed or mapped with low coverage by SGS reads. SMS isoforms showed more important merits in their sequence continuity. cDNA normalization strengthened these advantages of current SMS technology.

### cDNA normalization facilitates isoform phasing

Human is a diploid organism, and therefore it would be interesting to know whether a haplotype gene inherited from either parent transcribes and its relative transcript abundance. Single-nucleotide polymorphisms (SNPs) are often used for distinguishing the haplotype molecules, by a process called haplotying, i.e., to discern the parental origin of a single molecule based on the composition and connectivity of SNPs presented in the molecule. Long reads of SMS have been used for phasing isoforms^11,16^. Here, we also compared the capability of cDNA normalization in identification of allele-specific isoforms. The principles and performance of the phasing tool, ASIIQT, referred to Methods and Supplementary Fig. 5. The raw phasing data were stored in http://www.szu-bioinf.org/ASIIQT.

**Fig. 6:**
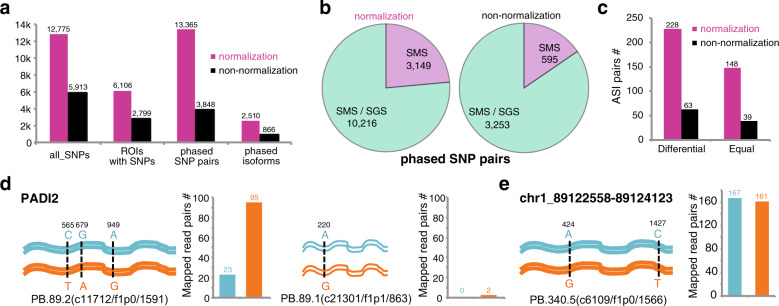
Effect comparison between cDNA-normalized and non-normalized SMS in phasing isoforms. **a** Basic statistics of isoform phasing for normalized and non-normalized SMS sequences. **b** Subgroups of SNP pairs phased by SMS reads. SMS: SNP pairs uniquely phased by SMS reads; SMS/SGS: SNP pairs phased by both SMS and SGS reads. **c** Allele-specific isoform (ASI) pairs with differential or equal expression level between alleles. **d** An example isoform of *PADI2* gene (PB.89.2) in the normalized libraries with ASI pairs that were differentially expressed (left). Another *PADI2* isoform (PB.89.1) was also phased, but the isoform was expressed lowly (right). **e** An example new gene with ASI pairs that were identically expressed in the normalized libraries. Data of human peripheral blood samples were used for the analysis.

More than twofolds of SNPs were called and covered by the ROIs of normalized libraries compared to non-normalized libraries in blood samples (12,775 vs 5913; Fig. 6a). The number of SNP-bearing ROIs was also much larger in the normalized libraries (6106 vs 2799; Fig. 6a). Accordingly, much more SNP pairs and isoforms were phased in the normalized libraries (13,365 vs 3848 and 2510 vs 866 for SNP pairs and isoforms, respectively; Fig. 6a). In normalized libraries, ~23.6% (3149/13,365) of the SNP pairs could not be phased by SGS reads owing to the short sequences. However, in non-normalized libraries, the percentage decreased to 15.5% (595/3848) (Fig. 6b). In total, 228 full-length high-quality isoforms were differentially expressed for the two alleles (≥30 total mapped SGS read pairs and greater than equal to twofold change), whereas 148 were equally expressed (≥30 total mapped read pairs and 1–1.105-fold change) in normalized libraries (Fig. 6c). In non-normalized libraries, the number of differentially and equally expressed allele-specific isoform pairs decreased to 63 and 39, respectively (Fig. 6c). We selected Peptidyl arginine deiminase 2 gene (*PADI2*) as an example for which three different isoforms were disclosed from normalized libraries. Two *PADI2* isoforms were also specifically detected using SGS reads, but only P.89.2 (c11712/f1p0/1591) showed high expression. Three SNPs in P.89.2 having a whole 384-nt span were phased, and the two alleles expressed differentially (Fig. 6d). In another example, we recognized a new transcript with only one exon (PB.340.5), for which two SNPs were called with >1 kb distance. This isoform was expressed abundantly, with equal level transcribed from both alleles (Fig. 6e).

### Full-length transcriptome of SRCCs

We extended the normalized single-molecule RNA-sequencing protocol to profile the transcriptome of SRCCs. Gastric cancer is one of the leading malignant tumors and causes of tumor death worldwide, especially in East Asia^17,18^. Although a majority of gastric cancers are adenocarcinomas, their genetics show high variance, and few genetic risks have been recognized as associated markers^19,20^. SRCC represents a specific type of gastric adenocarcinoma, which often shows higher malignancy, increasing incidence and higher mortality. The transcriptomes of gastric cancer, especially SRCC, remain under-explored.

**Fig. 7:**
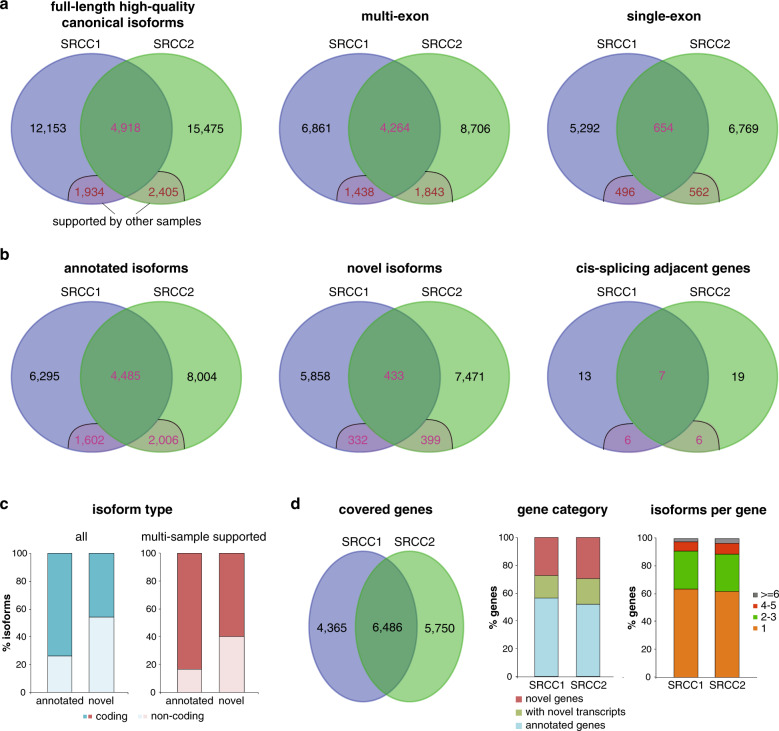
cDNA normalization based SRCC transcriptomes. **a** Summary of full-length high-quality isoforms identified from SRCCs. **b** Annotated and novel isoforms in SRCCs. **c** Percentages of coding and non-coding isoforms in SRCCs. **d** Genes covered by the isoforms detected from SRCCs. Genes detected from individual or both SRCC samples, gene category, and composition of genes based on isoform number were shown.

In total, 36,885 unique full-length high-quality canonical isoform clusters were obtained from SRCC samples, among which 4918 (13.3%) were shared by both tumors and 32–36% from each sample were supported by at least one other tumor or non-malignant sample (Fig. 7a; Supplementary Data 2; Supplementary Data 3). Multi-exon and single-exon isoforms covered ~63% and ~37% of the total, whereas ~61% and ~39% of the isoforms were annotated and novel ones, respectively (Fig. 7b; Supplementary Data 4). Like single-exon isoforms, the novel isoforms also showed larger variability from sample to sample, with only 8% being simultaneously supported by other samples, compared with 36% for annotated isoforms (Fig. 7b; Supplementary Data set 1). In total, 1164 isoform clusters were newly and repeatedly identified from this study, not annotated in the GENECODE database. We also identified 51 cis-splicing adjacent gene fusions, 19 among which were repeatedly identified from multiple samples (Fig. 7b; Supplementary Data 2). In all, 74% and 46% of the total multi-exon and single-exon isoforms showed protein encoding potentiality, respectively, and the percentages increased for the isoforms supported by more samples (Fig. 7c; Supplementary Data 2). The isoforms captured in SRCC1 and SRCC2 represented 10,851 and 12,236 genes, of which averagely 54%, 18%, and 28% were annotated genes with all annotated transcripts, annotated genes with novel transcripts and novel genes, respectively (Fig. 7d; Supplementary Data 2). Single isoforms were identified for ~63% of the genes, 2–3 isoforms were identified for ~27% of the genes, and only 10% of the genes were detected with four or more isoforms per gene (Fig. 7d; Supplementary Data 2).

**Fig. 8:**
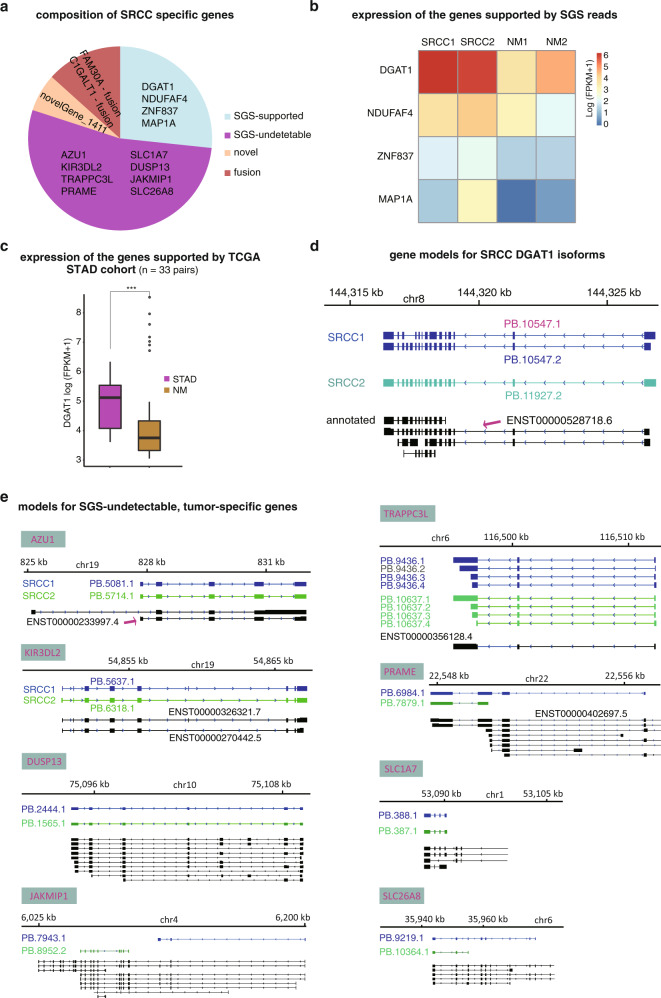
Genes specifically detected from the cDNA-normalized libraries of SRCCs compared with those of non-malignant gastric mucosa samples. **a** Gene specifically detected from both the SRCC samples. **b** Expression of the SGS supported genes. Gene quantification was based on SGS results. **c** The expression of an example (*DGAT1*) originated from TCGA STAD RNA-seq data source in the tumors and paired control samples. Mann–Whitney *U* test was performed. **d** Gene models for *DGAT1* isoforms. Isoforms from SRCC1, SRCC2, and GENECODE database were shown in blue, green and black, respectively. The database-annotated transcript corresponding to the SRCC-captured isoforms were indicated with arrow. The novel isoform detected from SRCC1 was shown in red. **e** The models for eight SGS-undetectable genes with isoforms specifically detected from both the SRCC samples. For all the statistical tests, *p < 0.05; **p < 0.01; ***p < 0.005.

The isoforms were compared between tumors and non-malignant pairs to identify SRCC-specific transcriptome signatures. In total, 15 multi-exon isoform bearing genes were identified exclusively from both SRCC specimens, including four annotated genes also detected by SGS short reads, eight annotated genes undetectable by SGS, two cis-splicing adjacent genes, and one novel gene (Fig. 8a). For the four SGS-detected genes, short-read quantification all confirmed higher expression in SRCC, and Diacylglycerol acyltransferase I (*DGAT1*) appeared most typical (Fig. 8b). SGS data for 33 pairs of stomach adenocarcinomas (STADs) and adjacent non-malignant tissues from The Cancer Genome Atlas (TCGA) cohort also demonstrated higher expression of *DGAT1* in cancers (Fig. 8c; Mann–Whitney *U* test, *p* = 0.002). A common isoform was found in both SRCC tissues (ENST00000528718.6, with slight difference at the 5′-end but no influence on the protein-coding frame) (Fig. 8d). A novel isoform (PB.10547.1) was also identified from one of the tumors (SRCC1) (Fig. 8d). cDNA-normalized SMS also disclosed the isoform composition of other SGS-detected genes, the novel gene and fusion genes, as could hardly be assembled or distinguished by SGS data (Supplementary Fig. 6). For the genes specifically detected from SMS libraries of both SRCCs but undetectable by SGS data, most of them, e.g., Azurocidin 1 (*AZU1*), Trafficking protein particle complex 3 like (*TRAPPC3L*), Dual specificity phosphatase 13 (*DUSP13*), Solute carrier family 1 member 7 (*SLC1A7*) and Killer cell immunoglobulin-like receptor 3DL2 (*KIR3DL2*), showed consistent transcript structure between the two tumors and/or had the transcripts with the same complete structure with annotated transcripts (Fig. 8e). Among them, two genes, preferentially expressed antigen in melanoma (*PRAME*) and Janus kinase and microtubule interacting protein 1 (*JAKMIP1*), were reported as cancer/testis (CT) genes that were selectively expressed in testis and cancers^21,22^. *DUSP13*, *TRAPPC3L,* and solute carrier family 26 member 8 (*SLC26A8*) were also selectively or highly expressed in testis and therefore could represent new CT genes^23–26^. *AZU1* and *KIR3DL2* were potentially related with tumor immune microenvironment^27,28^.

**Fig. 9:**
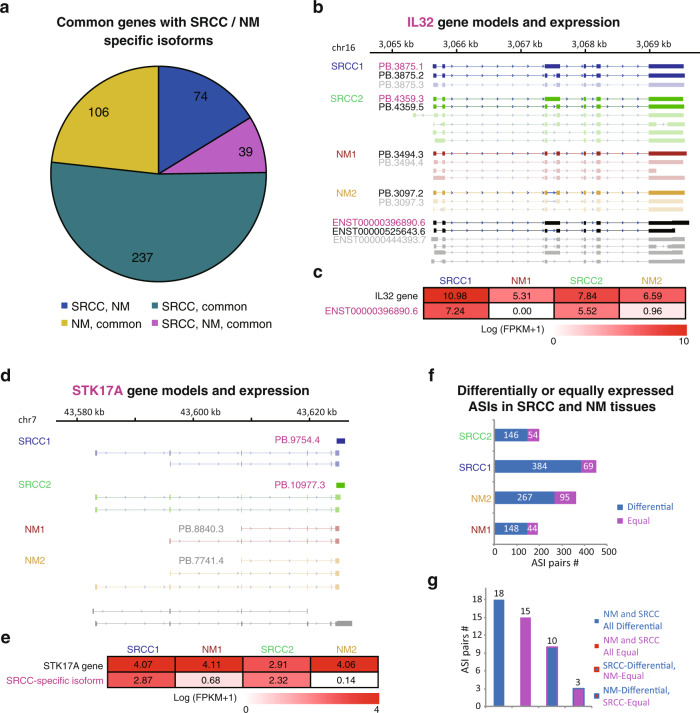
Isoforms specifically detected from the cDNA-normalized libraries of SRCCs compared with those of non-malignant gastric mucosa samples. **a** SRCC/non-malignancy (NM) common genes with SRCC, NM, or both specific isoforms. There were four groups, including those with both SRCC-specific and NM-specific isoforms (‘SRCC, NM’), with common and SRCC-specific isoforms (‘SRCC, common’), with common and NM-specific isoforms (‘NM, common’) and with common, SRCC-specific, and NM-specific isoforms (‘SRCC, NM, common’). Common or SRCC/NM-specific isoforms were detected from all the four samples or detected from the two SRCC/NM samples, respectively. **b**, **c** Gene models **b** and gene/isoform expression quantification **c** for *IL32*. **d**, **e** Gene models **d** and gene/isoform expression quantification **e** for *STK17A*. The names of SRCC-specific and common isoforms were shown in magenta and black, respectively. Quantification for the genes and SRCC-specific isoforms was based on SGS data. **f** Expression difference of phased isoforms between alleles in SRCC and NM samples. **g** Biased or equal allele-specific expression patterns of phased isoforms in SRCC and NM samples.

There were 456 genes commonly expressed in all the SRCC and non-malignant samples but with SRCC, non-malignancy or both specific isoforms (Fig. 9a; Supplementary Data 4). Interleukin 32 (*IL32*) and serine/threonine kinase 17a (*STK17A*) were taken as two examples. *IL32* has one isoform, ENST00000525643.6, which was identified from all the tumor and non-malignant samples (Fig. 9b). Besides the common one, the tumors also had a specific isoform, ENST00000396890.6, which was detected only in the SRCCs (Fig. 9b). Quantification of the *IL32* gene demonstrated no difference between the tumor and non-malignant tissues, but the FPKMs specifically mapped to the SRCC-specific isoform (ENST00000396890.6) showed substantial difference (Fig. 9c). Similarly, *STK17A* had an SRCC-specific isoform (PB.9754.4/PB.10997.3) and an NM-specific one (PB.8840.3/PB.7741.4), both of which were novel (Fig. 9d). There was no quantification difference between tumor and non-malignant tissues for *STK17A* gene, but difference was detected for the SRCC-specific isoform (Fig. 9e). With TCGA data, despite no difference being detected at gene level, significant difference was found between STADs and the non-malignant pairs for both *IL32* (Mann–Whitney *U* test, *p* = 4e-7) and *STK17A* (*p* = 0.0006) (Supplementary Fig. 7). Moreover, the higher expression of both *IL32* and *STK17A* gene was found to be associated with worse prognosis of STAD (Kaplan–Meier overall survival analysis, log-rank tests, *p* = 0.12 and 0.013 for *IL32* and *STK17A*, respectively; Supplementary Fig. 7). It was unclear whether STADs and SRCCs expressed the same tumor-specific isoform.

We also applied the ASIIQT protocol to identify and quantify the allele-specific isoforms in tumors and non-malignant pairs. In total, 7270 isoforms were phased (Supplementary Data 4; http://www.szu-bioinf.org/ASIIQT). From each sample, 146–384 full-length high-quality isoforms were identified with allele expression preference, whereas 44–95 were evenly expressed (Fig. 9f; Supplementary Data 4). Among them, 15 isoforms were consistently expressed with equal or nearly equal level between alleles among all the four samples, whereas 18 isoforms always showed allele-biased expression (Fig. 9g). Ten isoforms showed allele-biased expression in at least one tumor but equal in its non-malignant pair, whereas three isoforms allele-equally expressed in at least one tumor but biased in its paired non-malignancy (Fig. 9g). Four of them and two additional isoforms showed a similar expression-shifting trend for alleles from non-malignancy to tumor between the two patients (Supplementary Fig. 8).

## Discussion

Single-molecule RNA-seq techniques generate extended read length that could cover the full length of isoforms. However, the diversity of captured isoforms is extremely limited. Although new platforms have been released that could improve the sequencing output, the more stable platforms, e.g., PacBio RS II, remain the major choices for many research groups for isoform sequencing at present^29,30^. In this research, we integrated a DSN-based cDNA normalization procedure before the preparation of SMS libraries, to improve the diversity of transcripts and identify more genes with moderate or low expression. One major concern for the normalization procedure is the completeness of sequenced transcripts. We did find a 3′-breakdown for the ROIs of normalized libraries (Fig. 3b). However, these 3′-incomplete ROIs were filtered for further clustering and other analysis of high-quality isoforms, which required the presence of 3′-ployA tails and 5′/3′-primers. The primers were added to cDNA molecules before normalization. Therefore, the 5′- and 3′- distance to the aTSSs and aTTSs of transcripts became similar between the high-quality transcripts of normalized and non-normalized libraries (Fig. 3d). Another underlying drawback of cDNA normalization is that it equalizes different species of transcripts at an expense of losing original information on relative quantification of the genes or isoforms. However, this can be done by adding a cost-effective short-read RNA-seq experiment for the same samples. We did not make size fragmentation in this study; in practice, however, we recommend this step for the normalized cDNA molecules before preparation of the size-selected SMS libraries (e.g., 1–2 kb, 2–3 kb, and >3 kb), to avoid the length distribution bias of SMS reads to the best and to capture larger transcript diversity.

There are also other strategies to enrich rare transcripts and improve the diversity of transcripts. For example, targeted sequencing has been widely adopted to couple with long-read RNA sequencing, and disclosed a large number of important transcripts that were out of detection by traditional SMS methods^8,31–33^. Compared with cDNA normalization, targeted sequencing showed stronger capability in improving the diversity of sequenced isoforms. However, normalization also had its own merits. First, cDNA normalization can be performed to any organism while targeted sequencing can only be applied to model organisms with genome sequenced. Second, selection of interesting genome regions is a prerequisite for targeted RNA sequencing^8,32,33^. cDNA normalization does not have such limitations, and therefore is suitable for more general transcriptome studies. Third, the procedure of cDNA normalization is pretty straightforward, not like targeted sequencing, which involves genome segment selection, probe design, and enrichment of targeted molecules^31^. cDNA normalization provides a new option, and consequently, researchers can select the method most suitable for their specific projects.

ASIIQT was developed in this study to phase the isoforms from cDNA-normalized libraries. Other tools can also phase SMS long sequences, including HapIso^16^ and IDP-ASE^11^. Both HapIso and IDP-ASE take ROIs as input and phase them without correction of the non-SNP nucleotides. In contrast, there are two options for ASIIQT: (1) taking ROIs as input like HapIso and IDP-ASE, but reporting phased reads with non-SNP nucleotides corrected by high-accurate SGS short reads, or (2) taking both ROIs and FL high-quality isoforms as input and reporting the phased isoforms rather than the ROIs. These phased reads with correction or phased high-quality isoforms could have more direct use for downstream analyses and applications. Direct comparison of ASIIQT with HapIso suggested that ASIIQT had higher sensitivity in SNP calling and higher accuracy in phasing the SNPs. Moreover, ASIIQT required fewer computational resources and could call and phase Indels. IDP-ASE uses both SMS and SGS reads to phase the transcripts, whereas ASIIQT uses SGS reads to call SNPs and phase the isoforms only based on SMS sequences. Therefore, IDP-ASE could have higher sensitivity. However, ASIIQT remains a good option, especially when high-quality and full-length isoform haplotypes are more desired.

As an example for application, we used cDNA-normalized SMS to analyze the transcriptome of SRCCs. In total, 36,885 non-redundant full-length high-quality isoforms were captured from SRCCs, 14,493 among which were novel and not annotated in the latest GENECODE database, representing 6372 new genes and 3202 annotated genes with novel transcripts (Fig. 7). It demonstrated another advantage of the cDNA-normalized SMS, i.e., capturing a large number of new FL HQ transcripts from biologically important samples. We also identified four SRCC-specifically high-expressed genes and 350 SRCC-specifically expressed isoforms, for which *DGAT1*, *IL32,* and *STK17A* were typical examples (Figs. 8–9). *DGAT1* encodes a key enzyme in lipogenesis, which was found increased in prostate cancer cells and could facilitate cell proliferation and migration^34^. *DGAT1* gene expression was increased in both SRCCs and STADs (TCGA cohort) than non-malignant pairs, suggesting its increased expression in stomach adenocarcinoma universally. IL32 has been reported to relate with carcinogenesis, metastasis, angiogenesis, and regulation of the antitumor immune response in various tumors^35^. STK17A is a ubiquitously expressed kinase, which was reported to regulate apoptosis, epithelial phenotypes, etc., and showed suppression on tumor progression and invasion in several other tumors^36^. Neither *IL32* nor *STK17A* showed apparent expression difference at gene level but both showed SRCC-specific isoforms, for which SGS short-read quantification also supported the substantial increase of expression in SRCCs at the isoform level (Fig. 9b–e). TCGA data, however, reflected the increased expression of both *IL32* and *STK17A* in STADs compared with non-malignant pairs at gene level, and the potential association of their expression with STAD prognosis. It should be noted that the expression patterns and prognostic association of *STK17A* in STADs were in contrast to other tumors^36^.

With the cDNA-normalized SMS, we also found eight genes specifically expressed in SRCCs, which were undetectable from SGS libraries (Fig. 8e). Interestingly, five of them could potentially represent new CT genes in gastric cancers and two others were related with immune microenvironment. ASIIQT also identified a list of isoforms with allele-specific expression difference between SRCCs and non-malignant pairs (Fig. 9f–g). Taking together, we did not give an approach analyzing isoforms, but also provided valuable resources for transcriptomics/bioinformatics researchers with several high-quality bench-marking datasets with normalized and non-normalized SMS and paralleled SGS short-read data for multiple biological samples, and for research community of gastric cancer with SRCC transcriptome data, a number of SRCC-specific isoform candidates, highly or lowly expressed isoforms and allele-specific isoforms.

## Methods

### Human samples

In total, 20 ml fresh peripheral blood was collected from a healthy male subject. Two pairs of fresh gastric SRCC and para-carcinoma tissues derived from gastrectomy specimens were collected at the Department of Gastrointestinal Surgery of Shenzhen People’s Hospital (Shenzhen, China) between April 2018 and June 2018 in this study. Tumor samples were re-assessed by two pathologists and the percentage of tumor cells was 80% or more. The majority (>70%) of the tumor cells were signet-ring cells under microscopy. None of the patients received chemotherapy or radiotherapy before surgical resection. This study was approved by the Human Ethics Approval Committee at Shenzhen People’s Hospital. Written informed consents for their samples to be used in a future study were obtained from all patients or healthy subjects.

### RNA extraction and full-length cDNA synthesis

Total RNA was isolated using QIAamp RNA Kit (QIAGEN catalog #52304) for blood samples or TRIzol reagent (Invitrogen™ catalog #15596-026) for solid samples. The quality of total RNA was assessed using a Bioanalyzer 2100 (Agilent Technologies, USA). The first-strand cDNA was synthesized with SMARTer PCR cDNA Synthesis Kit (Clontech catalog #634925) and the 3′ SMART CDS Primer IIA (5′-AAGCAGTGGTATCAACGCAGAGTACT(_30_)N_−1_N-3′), according to the manufacturer’s protocol. The second-strand cDNA was synthesized and the product was further amplified with PrimeSTAR HS DNA Polymerase (TAKARA catalog #R010A) and Primer M1 (5′-AAGCAGTGGTATCAACGCAGAGT-3′) for 12–15 PCR cycles (15 s at 95 °C, 30 s at 65 °C, and 6 min at 68 °C). cDNA product was purified with TaKaRa MiniBEST DNA Fragment Purification Kit (Catalog #9761) and dissolved with sterile RNAse-free water to a final concentration of 100 ng/μl.

### Second-generation RNA sequencing and data processing

The full-length cDNA molecules were fragmentized and used for preparation of Illumina TruSeq RNA libraries with an insert size of 300–400 bp. Each library was verified for the size with Bioanalyzer 2100 and quantified according to Illumina qPCR Quantification Protocol Guide (Illumina, USA). Illumina HiSeq 2000 250PE (250-bp read length, paired-end) platform was used for human peripheral blood, and HiSeq 4000 250PE was used for tumor and paired non-malignant samples. After the adapters and low-quality reads were filtered, the Bowtie-TopHat-Cufflinks pipeline was applied to map the filtered reads to human reference genome and quantify the expression of genes or isoforms^37^. The NCBI build 38 (GRCh38) of hg38 and GENECODE version 31 were used^38^. To align the reads against long transcript sequences, bowtie was used, and SAM files were generated for further analysis.

### cDNA normalization

DSN-based cDNA normalization referred to the protocol of Zhulidov 2004^13^, but was performed to double-strand full-length cDNA, and no size fractionation was performed before normalization. Normalization was performed with DSN (Evrogen Trimmer-2, catalog #NK003), according to the product manual with few modifications for optimization especially for the enzyme concentration and normalization period. In brief, 10 μl of the cDNA solution (~1 μg cDNA) was mixed with 4 μl 4× hybridization buffer (200 mM Hepes, pH 7.5, 2 M NaCl) and 2 μl sterile RNase-free water, overlaid with a drop of mineral oil, denatured at 98 °C for 2 min, and then incubated at 68 °C for 5 h. In all, 5 μl preheated DSN master buffer (100 mM Tris-HCl, pH8.0, 10 mM MgCl2, 2 mM DTT) was added to the reaction mixture and incubated at 68 °C for 10 min, followed by adding 0.25 U/μl of DSN enzyme and incubation in a thermal cycler at 68 °C for 25 min. In all, 5 μl 10 mM ethylenediaminetetraacetic acid (EDTA) was subsequently used to inactivated the DSN. After DSN inactivation, sterile RNase-free water was added to the reaction mixture to a final volume of 40 μl. An aliquot (1 μl) of the DSN-treated solution was used for PCR with 0.3 μM 5′ Primer M1 in a 50 μl reaction volume containing 1.25 U PrimeSTAR HS DNA Polymerase (TAKARA catalog #R010A), 5× PrimeSTAR buffer and 100 μM dNTP mixture. In all, 12–15 PCR cycles were performed. The normalized cDNA product was purified with TaKaRa MiniBEST DNA Fragment Purification Kit. Gel electrophoresis was performed to evaluate the effect of normalization. The concentration of DSN enzyme and incubation period should be adjusted and optimized for different RNA samples and determined based on the gel electrophoresis results. After purification, the normalized cDNA products could be used for SMS library preparation and sequencing directly. The cDNA-normalized products could be size-fractionized and amplified for each fraction optionally (normally three fractions of 1–2 kb, 2–3 kb, and >3 kb, or four fractions of 1–2 kb, 2–3 kb, 3–6 kb, >5 kb).

### SMS library preparation, sequencing, and data preprocessing

Libraries of ~20 kb SMRTbell Templates were prepared for the DSN-treated (normalized) or non-treated cDNA. Libraries were checked for the quality and quantified, and sequenced using SMRT sequencing on the PacBio RS II System (Pacific Biosciences, USA). For each sample, normalized or non-normalized, two cells were sequenced. ROIs were called and corrected from the raw polymerase reads with SMRT analysis 2.3.0 (Pacific Biosciences, USA). More than one full pass was required to build the ROIs. The library preparation, sequencing and preprocessing of raw data were performed in Macrogen, Korea. SMRT Portal IsoSeq analysis was further performed with default parameters to filter the artifacts, classify and cluster the ROIs to obtain the full-length reads, consensus isoforms and high-quality isoforms respectively, which were used for further analysis. The criteria of consensus isoforms and high-quality isoforms conformed to PacBio SMRTLink default definition, that is, a consensus isoform should contain at least one full-length ROI (with 3′-polyA tail, 5′- and 3′-primers) and the predicted accuracy of sequences reached 95–99%, whereas a high-quality isoform should be supported by at least two full-length ROIs and the predicted sequence accuracy should not be lower than 99%.

### Transcript collapsing and annotation

Cupcake ToFU version 8.5 was used to further collapse the high-quality isoforms and filter 5′-degraded isoforms (https://github.com/Magdoll/cDNA_Cupcake/). The generated unique full-length high-quality isoforms were aligned against human reference genome hg38 with GMAP^39^, with parameters of “-B 2 -f samse–chimera-margin 30 -n 5–min-intronlength 25 -z sense_force”. Transcripts mapped to multiple genome loci were filtered using the similar criteria adopted by Tilgner et al.^40^. The alignment results were based on to parse the structure of uniquely mapped transcripts, according to which a transcript was classified into single-exon or multi-exon isoform. For each ME isoform, the nucleotides of splicing sites were retrieved and examined whether they were canonical with intron edges of ‘GT-AG’, ‘GC-AG’, or ‘AT-AC’. An ME isoform was further categorized as ME_canonical if it contained all canonical splicing sites, or ME_non-canonical if there was at least one non-canonical splicing site. Transcripts were assigned to genes and known transcripts according to the genome annotation of GENECODE version 31. Canonical splicing sites from the full-length high-quality isoforms were checked with the splicing sites of annotated transcripts so as to identify the validated ones. A ME_canonical isoform was further classified as a CSSM, Non-CSSM or Novel one based on the alignment results and known structure of transcripts annotated in GENECODE. A CSMM was attributed to a spliced gene if they shared at least one splice site. If multiple genes had the common splice sites with a CSMM, the one with which it shared most common splice sites was attributed to ref. ^6^. A non-CSSM was an isoform that shared no splice site with the assigned gene. A novel isoform could not be assigned to any known gene. A molecule was attributed to an annotated spliced transcript, when the molecule showed common composition and structure (donor and receptor sites) for all the introns contained by both of them. Two molecules were attributed to a same transcript if they shared the same composition and structure for all introns and no larger than 200-nt difference for the 5′- or 3′-ends. SQANTI was also applied to further annotate the ToFU collapsed isoforms of tumor and paired non-malignant gastric samples^41^.

### Analysis of the completeness of transcripts

All the annotated transcripts in GENECODE version 31 were retrieved, and the ones were selected for further analysis that encode proteins or belong to processed transcripts, retained introns, nonsense mediated decays, processed pseudogenes, and lncRNAs^6,38^. For each annotated transcript, the aTSS was defined as the first nucleotide in its transcription direction, whereas the aTTS was defined as the last nucleotide in the transcription direction. The distance of a CSMM from an aTSS or aTTS was defined and calculated according to ref. ^6^. In brief, the distance of a CSMM from an aTSS was calculated as the minimal downstream distance between the start site of the CSMM and all aTSSs of the transcripts that belong to the CSMM-attributed gene and have the same transcription direction with the CSMM. Similarly, the distance of a CSMM from an aTTS was calculated as the minimal upstream distance between the termination site of the CSMM and all aTTSs of the transcripts that belong to the CSMM-attributed gene and have the same transcription direction with the CSMM. All the CSMMs with negative distance to aTSSs or aTTSs were considered full-length and the distance was recorded as zero.

### Gene and isoform quantification with SGS reads

The Bowtie-TopHat-Cufflinks pipeline was used to map the SGS reads to human genome hg38 and quantify the expression of genes^37^. The isoforms detected with SMS were mapped to human genome with GMAP^39^, and clustered for each gene unit according to mapping results. For different isoforms of a gene, the unique exon–exon junction sites and exon regions were retrieved. The SGS reads from the same sample were mapped to reference genome with bowtie. Read pairs were counted that specifically mapped to the unique exon–exon junction sites and exon regions for each isoform. DESeq2 was used to normalize and compare the expression of isoforms^40^.

### Phasing and quantification of allele-specific isoforms

A new tool, ASIIQT, was developed to specifically phase and quantify the isoforms captured by cDNA-normalized SMS and paralleled SGS. The pipeline of ASIIQT was shown in Supplementary Fig S5. Uncorrected ROI reads and paired SGS reads were used for SNP calling and phasing. ROI reads were mapped to human genome with GMAP and the resulting SAM file was based on to build pseudo-long reads with the ROI-derived isoform frames and reference genome replaced nucleotides. BWA was used to map the SGS reads to pseudo-long reads, and SAMtools was used to identify the possible SNPs^42,43^. The non-SNP positions within a pseudo-long read mapped by ≥2 SGS reads without inconsistency of nucleotide composition were replaced with the nucleotides present in SGS reads. All the possible SNP positions within any pseudo-long read were marked, whereas those with homozygous nucleotide composition in SGS reads but different from the composition in the pseudo-long read were replaced with corresponding nucleotides in SGS reads. The remaining SNP positions (marked) and SGS-uncovered regions were replaced with corresponding nucleotide composition in original ROIs, generating SGS-corrected ROIs. The nucleotide composition in each SNP position of an SGS-corrected ROI was compared with the SNP set called with SGS short reads, fixed if the composition was consistent, and phased if the composition of more than one SNP positions was fixed in one ROI. Both the SGS-derived SNP set and the obtained linkage of SNP composition in a subset of ROIs were referred and further applied to other ROIs, to correct possible sequencing errors in SNP positions and phase more ROI sequences. The isoform specifically mapped SGS reads were further divided into two alleles according to the SNP identification and phasing results, and by this way the expression of allele-specific isoforms was quantified. ASIIQT pipeline was developed with GO programming language, which is accessible through the link: http://www.szu-bioinf.org/ASIIQT, together with the introduction and manual. The performance of ASIIQT was evaluated and compared with HapIso^16^, an isoform-phasing tool that also mainly relies on SMS long reads. Generally, ASIIQT showed a high accuracy and much higher sensitivity in phasing compared with HapIso (http://www.szu-bioinf.org/ASIIQT/performance.html).

### Real-time PCR

Ambion™ External RNA Controls Consortium (ERCC) RNA Spike-in Mixes (Thermo Fisher Scientific catalog #4456740) were loaded (1:100) into the total RNA extracted from blood samples. Full-length cDNA synthesis and/or cDNA normalization were performed as described before. Three ERCC molecules were selected for relative expression examination using real-time quantitative PCR (qPCR), including ERCC-00002, ERCC-00113 and ERCC-00076. Primers for qPCR were designed based on the sequences of ERCC molecules (Thermo Fisher Scientific) and synthesized. 1 µl of the non-normalized or normalized cDNA was used as template. The relative expression levels of genes were detected with an ABI 7900 system (Applied Biosystems) using SYBR green reagent (Applied Biosystems). Each sample was assayed in triplicate.

### STAD RNA-seq data and survival analysis

The FPKM quantification results of RNA-seq data for STAD and partially paired (32) normal gastric mucosa samples were downloaded from TCGA^19^. Mann–Whitney *U* tests were performed to compare gene quantification results based on the 32 pairs of STAD and non-malignant samples. The clinical follow-up data were also downloaded from TCGA, and the patients that received targeted therapies were removed for survival analysis. For each interesting gene, the expression in tumors of the remained patients was noted down, and the median was used as the cutoff value. The cases were stratified into two groups, one with tumors of lower expression for the corresponding gene (<cutoff value) and the other with tumors of higher expression (≥cutoff value). The Kaplan–Meier overall survival curves were compared between the two groups, with log-rank tests performed to test the significance of difference. For all statistical comparisons, the significance level was preset as *p* < 0.05.

### Statistics and reproducibility

The cDNA normalized and non-normalized SMS libraries were built for one replicate for each sample, and no statistic tests were performed between normalized and non-normalized groups within or among biological samples. Boxplots were made for the expression of all captured genes or indicated subsets of genes, and Mann–Whitney *U* tests were performed to compare gene quantification results. Survival analysis and other statistic analysis were indicated and described in other Methods sections and context. Significance level was preset as *p* < 0.05.

### Reporting summary

Further information on experimental design is available in the Nature Research Reporting Summary linked to this paper.

## Supplementary information

Supplementary Information

Supplementary Data 1

Supplementary Data 2

Supplementary Data 3

Supplementary Data 4

Reporting Summary

Description of Additional Supplementary Files

## Data Availability

The non-normalized and normalized SMS and SGS data for human peripheral blood, gastric signet cell carcinomas and paired non-malignant tissues were submitted to the SRA database, which could be accessed through the accessions SRR8668488-SRR8668502. The phased isoform sequences and related information were stored and are accessible without constraint at http://www.szu-bioinf.org/ASIIQT.

## Code availability

The codes for ASIIQT are available freely through the link: http://www.szu-bioinf.org/ASIIQT/ASIIQT.v.1.0.tar.gz. It could be also assessed via Zenodo: 10.5281/zenodo.3901013.
