# Supplementary material for: Improving the diversity of captured full-length isoforms using a normalized single-molecule RNA-sequencing method
Source: Commun Biol. 2020 Jul 30;3:403. doi: 10.1038/s42003-020-01125-7 (PMC7393167; doi:10.1038/s42003-020-01125-7)
Supplement: Supplementary file 1 — Supplementary Information [file 42003_2020_1125_MOESM1_ESM.pdf]

Improving the diversity of captured full-length isoforms using a normalized single-molecule RNA sequencing method

SUPPLEMENTARY INFORMATION

Supplementary figures

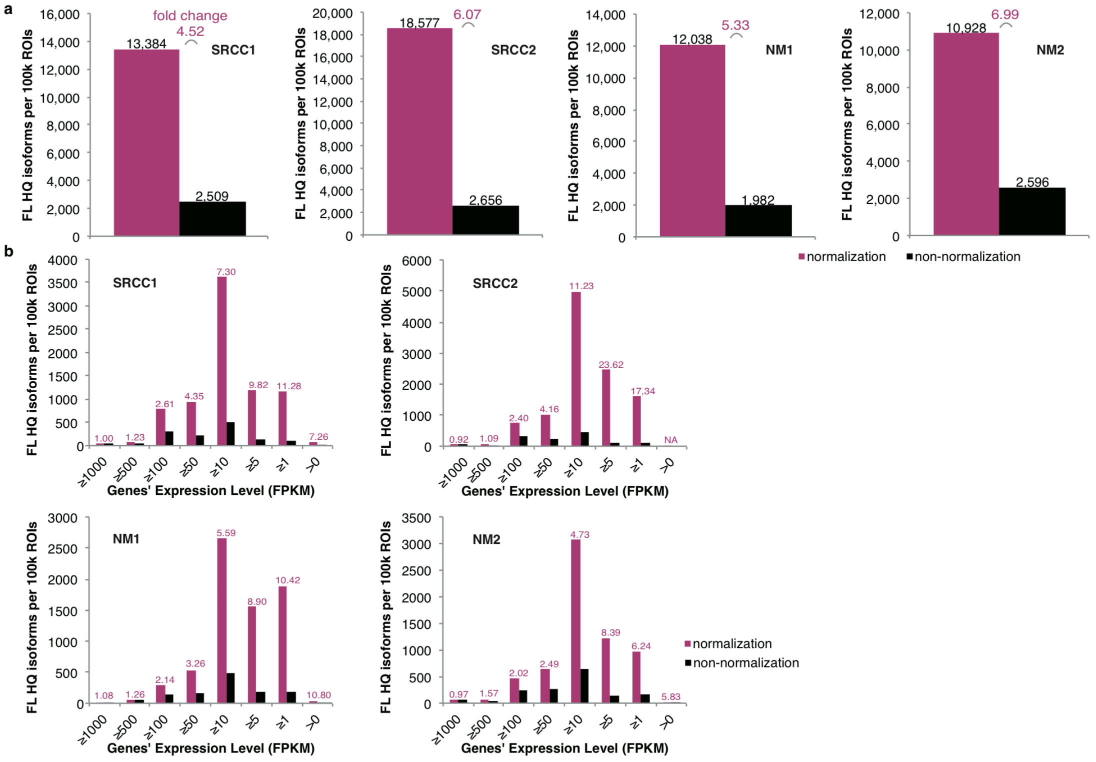

Supplementary Figure 1. Effect comparison between cDNA normalized and non-normalized SMS in diversity of isoforms captured from SRCCs and their adjacent non-malignant (NM) samples. (a) Overall difference in the diversity of captured full-length high-quality isoforms in two tumors (SRCC1 and SRCC2) and two non-malignant samples (NM1 and NM2). **(b)** Difference in the diversity of captured isoforms with varied gene expression levels in SRCC and non-malignant (NM) samples. The isoform-represented genes were binned according to their expression in each sample as measured by FPKMs of Illumina SGS data. Fold changes between the isoform counts in the normalized libraries vs. non-normalized libraries were indicated on the top of bars.

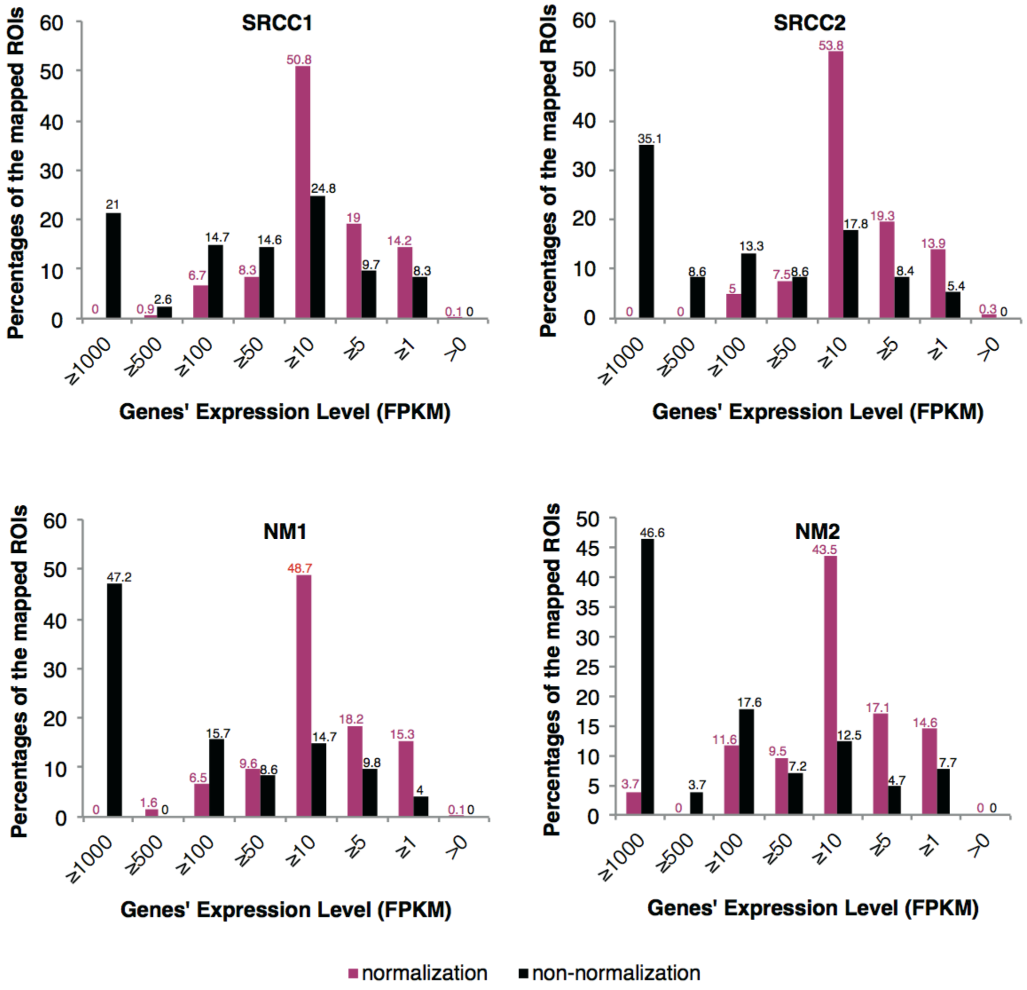

Supplementary Figure 2. Occupancy percentages of ROIs for the full-length high-quality isoforms with varied expression captured from SRCCs and non-malignant (NM) samples. The isoform-represented genes were binned according to their expression in each sample as measured by FPKM of Illumina SGS data.

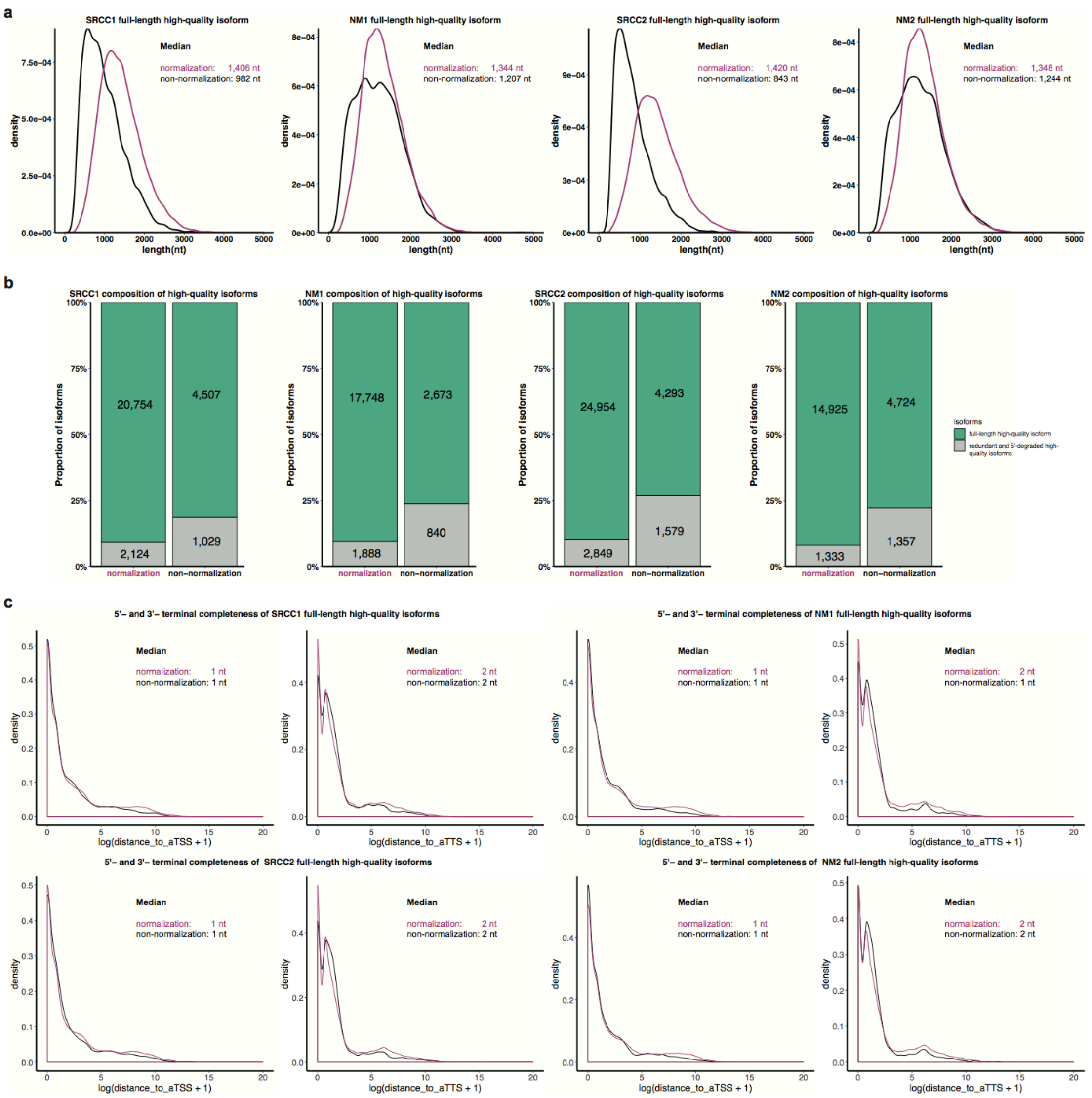

Supplementary Figure 3. Effect comparison between cDNA normalized and non-normalized SMS in completeness of isoforms captured SRCCs and non-malignant (NM) samples. (a) Length distribution of full-length high-quality isoforms. **(b)** The composition of high-quality isoforms. The high-quality isoforms retained as FL HQ ones were shown in green, while the filtered-away ones because of redundancy or 5'-degradation were shown in grey. **(c)** The distribution of distance to annotated TSSs (aTSS, left) and annotated TTSs (aTTS, right) for full-length

high-quality isoforms captured from each SRCC or NM sample. The normalized and non-normalized libraries were shown in magenta and black, respectively.

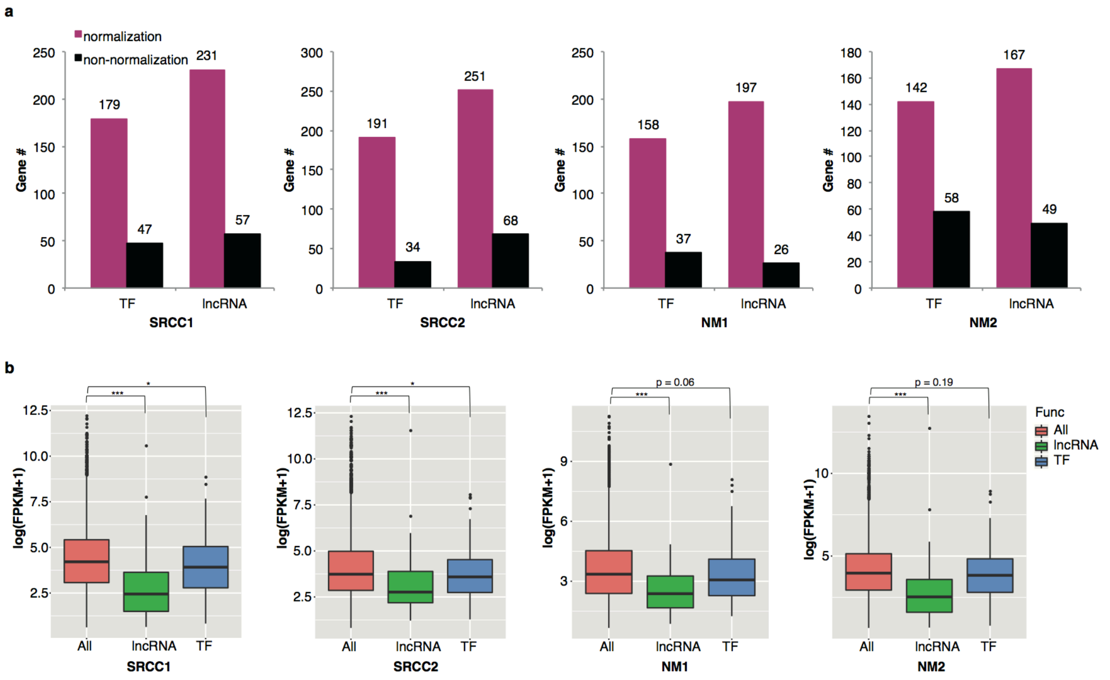

Supplementary Figure 4. Effect comparison between cDNA normalized and non-normalized SMS in detection of lncRNAs and TF genes from SRCCs and non-malignant (NM) samples. (a) Comparison of the lincRNAs and TF genes captured from each tumor or NM sample between normalized and non-normalized libraries. **(b)** Expression comparison between lncRNA / TF genes and all genes detected in normalized libraries of the tumor or NM samples. Mann–Whitney U tests were performed. Gene quantification was based on Illumina SGS results. For all the statistical tests, *, $p < 0.05$; **, $p < 0.01$; ***, $p < 0.005$.

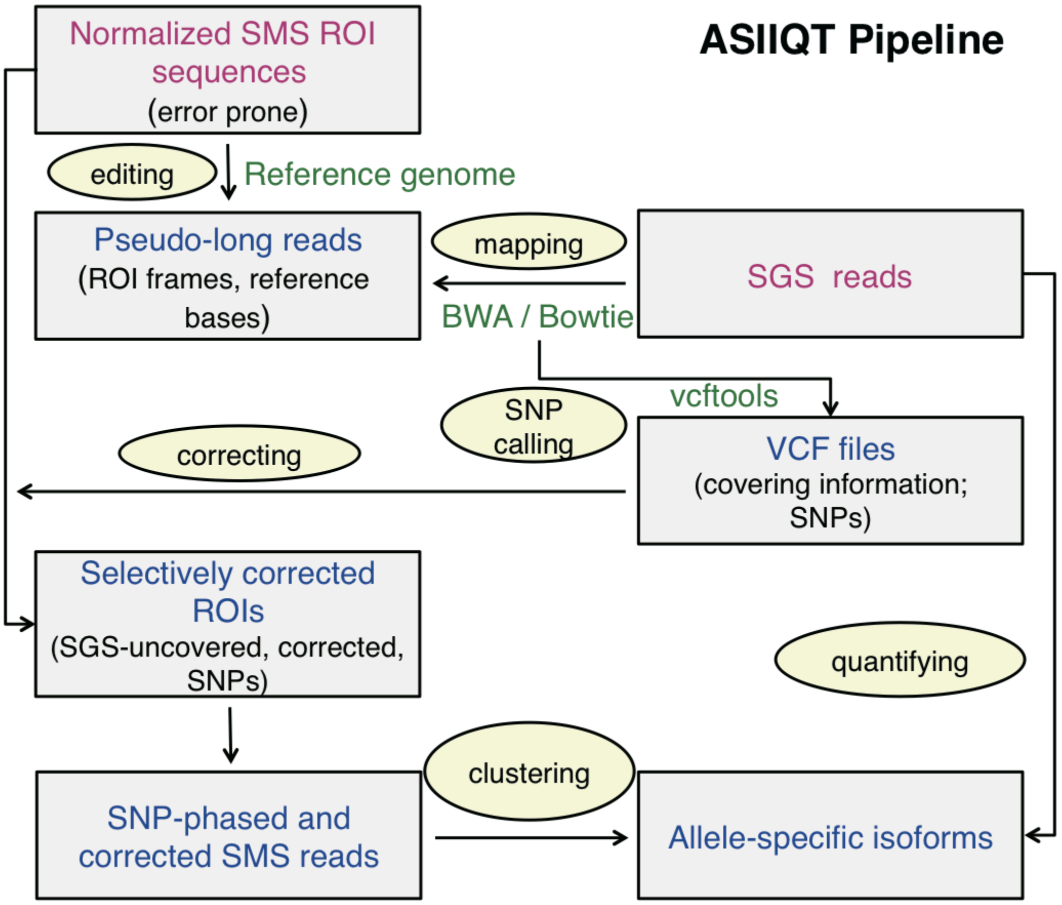

Supplementary Figure 5. The pipeline of ASIIQT. Pseudo-long reads were generated based on the alignment results of ROIs against the reference genome, with the frames and genome-mapped locations of ROIs but the nucleotides of the reference genome so as to eliminate the high errors of ROI sequences but retain the isoform structure. VCF files contained the SNPs and reference-covering information, i.e., genome loci covered by short reads. Selectively corrected ROIs were modified ROI sequences with SGS-covered non-SNP regions corrected.

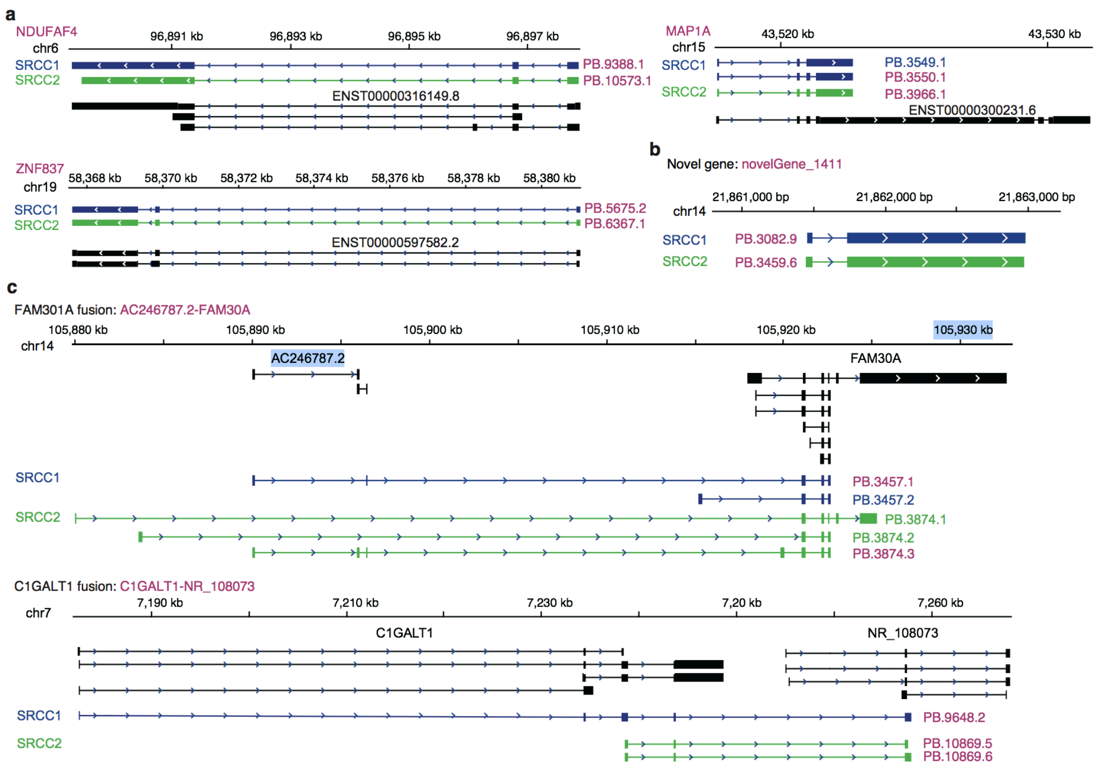

Supplementary Figure 6. The isoform composition and models of genes specifically detected from SRCCs with cDNA-normalized SMS. (a) Three genes supported by SGS short reads. **(b)** A novel gene identified by cDNA-normalized SMS. **(c)** Two fusion genes. For **(a)**, **(b)** and **(c)**, Gene models for SRCC1, SRCC2 and GENECODE annotated isoforms (if there were) were shown in blue, green and black, respectively. For **(a)** and **(b)**, the same isoforms in SRCC1 and SRCC2 were highlighted in magenta for the accessions and the annotated transcript corresponding to the isoforms (if there was) was also indicated. For **(c)**, the accessions of fusion isoforms were highlighted in magenta while non-fusions were shown in blue (SRCC1) or green (SRCC2).

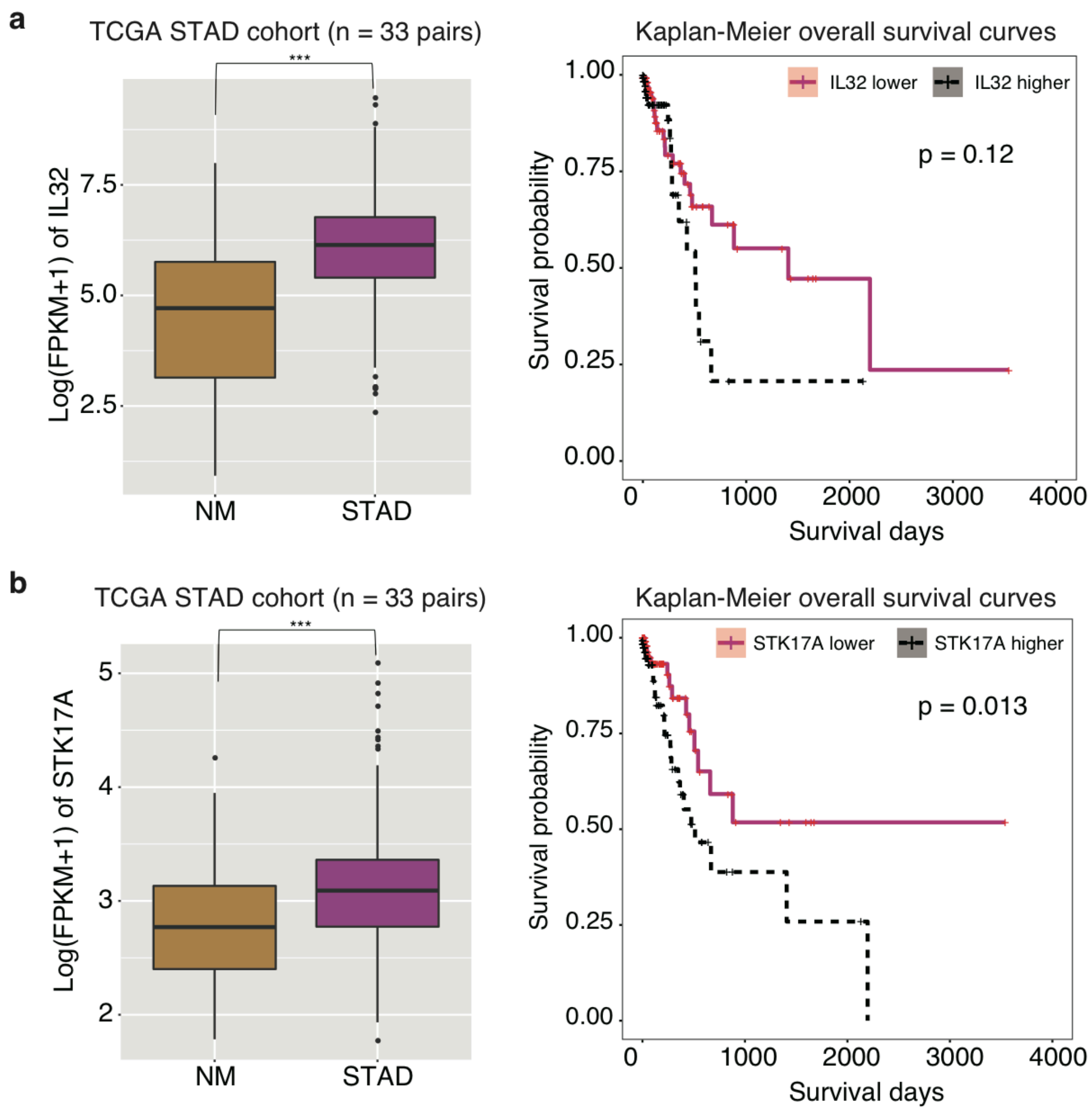

Supplementary Figure 7. Gene expression difference of IL32 and STK17A between TCGA STADs and their non-malignant pairs and the association of their expression with STAD overall prognosis. (a) IL32 gene expression difference and the Kaplan-Meier survival curves of STAD cases stratified by the IL32 expression. **(b)** STK17A gene expression difference and the Kaplan-Meier survival curves of STAD cases stratified by the STK17A expression. The cutoffs for gene expression stratification were medians, with '>=median' being 'higher' and '<median' being 'lower'. Mann–Whitney U tests were performed to compare gene quantification

results based on TCGA RNA-seq data. Log-rank tests were performed to compare the overall survival. For all the statistical tests, *, $p < 0.05$; **, $p < 0.01$; ***, $p < 0.005$.

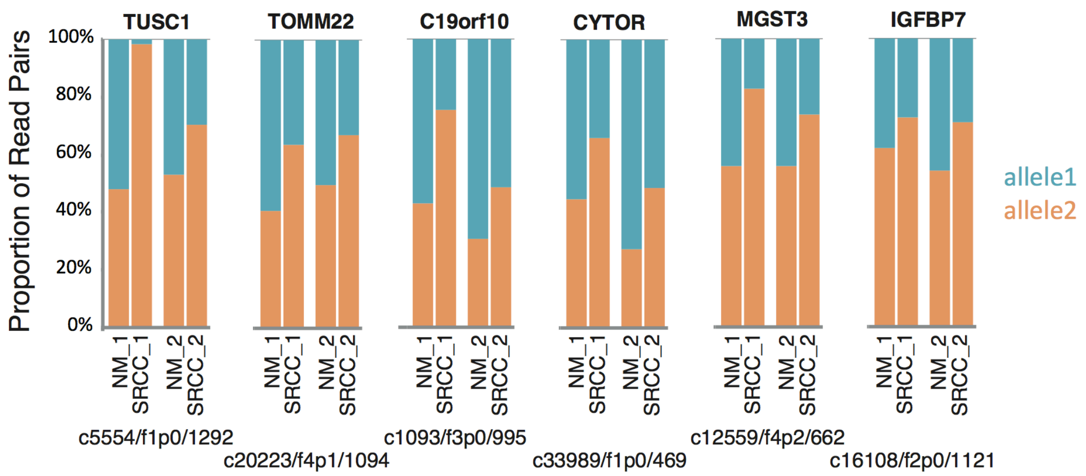

Supplementary Figure 8. Representative isoforms with allele-specific expression patterns shifting from non-malignancy (NM) to SRCC tissues consistently. Corresponding isoforms and represented genes were shown on the bottom and top of the bars respectively.

Supplementary tables

Supplementary Table 1. The splice junction patterns detected from each SMS library

Sample	Library	GT-AG (Percentages)	GC-AG (Percentages)	AT-AC (Percentages)	Others (Percentages)
PBMC	Normalized	49,326 (97.08)	410 (0.81)	90 (0.18)	979 (1.93)
PBMC	Non-normalized	8097 (96.18)	63 (0.75)	6 (0.07)	253 (3.00)
SRCC1	Normalized	82,354 (97.49)	666 (0.79)	128 (0.15)	1,328 (1.57)
SRCC1	Non-normalized	12,860 (96.88)	100 (0.75)	21 (0.16)	293 (2.21)
NM1	Normalized	68,512 (97.60)	477 (0.68)	115 (0.16)	1,094 (1.56)
NM1	Non-normalized	9,931 (97.52)	82 (0.81)	15 (0.15)	156 (1.53)
SRCC2	Normalized	97,293 (97.36)	836 (0.84)	138 (0.14)	1,663 (1.66)
SRCC2	Non-normalized	9,671 (96.61)	79 (0.79)	14 (0.14)	246 (2.46)
NM2	Normalized	58,230 (97.51)	464 (0.78)	89 (0.15)	934 (1.56)
NM2	Non-normalized	17,411 (96.93)	135 (0.75)	29 (0.16)	387 (2.15)
