# Supplementary material for: Improving the diversity of captured full-length isoforms using a normalized single-molecule RNA-sequencing method
Source: Commun Biol. 2020 Jul 30;3:403. doi: 10.1038/s42003-020-01125-7 (PMC7393167; doi:10.1038/s42003-020-01125-7)
Supplement: Supplementary file 6 — Reporting Summary [file 42003_2020_1125_MOESM6_ESM.pdf]

nature research

Corresponding author(s): Yejun Wang

Last updated by author(s): Jun 20, 2020

Reporting Summary

Nature Research wishes to improve the reproducibility of the work that we publish. This form provides structure for consistency and transparency in reporting. For further information on Nature Research policies, see Authors & Referees and the Editorial Policy Checklist.

Statistics

For all statistical analyses, confirm that the following items are present in the figure legend, table legend, main text, or Methods section.

n/a	Confirmed	
☐	☒	The exact sample size (*n*) for each experimental group/condition, given as a discrete number and unit of measurement
☐	☒	A statement on whether measurements were taken from distinct samples or whether the same sample was measured repeatedly
☐	☒	The statistical test(s) used AND whether they are one- or two-sided<br>*Only common tests should be described solely by name; describe more complex techniques in the Methods section.*
☐	☒	A description of all covariates tested
☐	☒	A description of any assumptions or corrections, such as tests of normality and adjustment for multiple comparisons
☐	☒	A full description of the statistical parameters including central tendency (e.g. means) or other basic estimates (e.g. regression coefficient) AND variation (e.g. standard deviation) or associated estimates of uncertainty (e.g. confidence intervals)
☐	☒	For null hypothesis testing, the test statistic (e.g. *F*, *t*, *r*) with confidence intervals, effect sizes, degrees of freedom and *P* value noted<br>*Give P values as exact values whenever suitable.*
☒	☐	For Bayesian analysis, information on the choice of priors and Markov chain Monte Carlo settings
☒	☐	For hierarchical and complex designs, identification of the appropriate level for tests and full reporting of outcomes
☒	☐	Estimates of effect sizes (e.g. Cohen's *d*, Pearson's *r*), indicating how they were calculated

Our web collection on statistics for biologists contains articles on many of the points above.

Software and code

Policy information about availability of computer code

Data collection	No software was used
Data analysis	Open-source tools: SMRT Analysis 2.3.0; GMAP v2017-11-15; Bowtie2; TopHat2; Cufflinks<br>New software tool developed in the study: http://www.szu-bioinf.org/ASIIQT

For manuscripts utilizing custom algorithms or software that are central to the research but not yet described in published literature, software must be made available to editors/reviewers. We strongly encourage code deposition in a community repository (e.g. GitHub). See the Nature Research guidelines for submitting code & software for further information.

Data

Policy information about availability of data

All manuscripts must include a data availability statement. This statement should provide the following information, where applicable:

- Accession codes, unique identifiers, or web links for publicly available datasets
- A list of figures that have associated raw data
- A description of any restrictions on data availability

Accessions: SRR8668488 - SRR8668502 (NCBI SRA database); Web link: http://www.szu-bioinf.org/ASIIQT

Field-specific reporting

Please select the one below that is the best fit for your research. If you are not sure, read the appropriate sections before making your selection.

☒ Life sciences ☐ Behavioural & social sciences ☐ Ecological, evolutionary & environmental sciences

Life sciences study design

All studies must disclose on these points even when the disclosure is negative.

Sample size	Samples: One blood, two gastric cancers and two non-malignant gastric tissues; two replicate libraries for each sample.<br>Rationale: We tried to observe and compare the effect of different protocols. Three different types of samples, and in total 5 individuals are sufficient, especially when the big cost is taken into consideration.
Data exclusions	No data were excluded from the analyses.
Replication	We compared the effect and verified the reproducibility of results in three types of samples with different origins. We confirm that all attempts at replication were successful.
Randomization	Randomization is not relevant to this study, because it is a paired design, i.e., different treatments for the same samples.
Blinding	The investigators were blinded to groups during the data collection and analysis.

Reporting for specific materials, systems and methods

We require information from authors about some types of materials, experimental systems and methods used in many studies. Here, indicate whether each material, system or method listed is relevant to your study. If you are not sure if a list item applies to your research, read the appropriate section before selecting a response.

Materials & experimental systems

n/a	Involved in the study	
☒	☐	Antibodies
☒	☐	Eukaryotic cell lines
☒	☐	Palaeontology
☒	☐	Animals and other organisms
☐	☒	Human research participants
☒	☐	Clinical data

Methods

n/a	Involved in the study	
☒	☐	ChIP-seq
☒	☐	Flow cytometry
☒	☐	MRI-based neuroimaging

Human research participants

Policy information about studies involving human research participants

Population characteristics	The samples of this research were collected from 1 healthy adult male subject and 2 adult female patients diagnosed with gastric signet-ring cell carcinoma, 28 years and older, all of whom are of Chinese Han ethnicity.
Recruitment	20 ml fresh peripheral blood was collected from a healthy male volunteer. Two pairs of fresh gastric signet-ring cell carcinoma and para-carcinoma tissues derived from gastrectomy specimens were collected at the Department of Gastrointestinal Surgery of Shenzhen People's Hospital (Shenzhen, China) between April 2018 and June 2018 in this study. Tumor samples were re-assessed by two pathologists and the percentage of tumor cells was 80% or more. The majority (>70%) of the tumor cells were signet-ring cells under microscopy. None of the patients received chemotherapy or radiotherapy before surgical resection.
Ethics oversight	All study procedures were approved by the Human Ethics Approval Committee at Shenzhen People's Hospital. Written informed consent was obtained from all participants

Note that full information on the approval of the study protocol must also be provided in the manuscript.
