# Supplementary material for: Improving the diversity of captured full-length isoforms using a normalized single-molecule RNA-sequencing method
Source: Commun Biol. 2020 Jul 30;3:403. doi: 10.1038/s42003-020-01125-7 (PMC7393167; doi:10.1038/s42003-020-01125-7)
Supplement: Supplementary file 7 — Description of Additional Supplementary Files [file 42003_2020_1125_MOESM7_ESM.pdf]

Description of additional supplementary files:

File Name: Supplementary Data 1

Description: Summary of PacBio (SMS) and Illumina (SGS) RNA-Seq data of human peripheral blood mononuclear cells (PBMC), gastric SRCCs and paired non-malignant (NM) gastric tissues.

File Name: Supplementary Data 2

Description: Full-length high-quality isoforms captured in the cDNA-normalized SMS libraries of SRCCs.

File Name: Supplementary Data 3

Description: Annotation of the full-length high-quality isoforms captured from the cDNA-normalized SMS libraries of SRCC and paired non-malignant gastric samples.

File Name: Supplementary Data 4

Description: SRCC- and NM- specific full-length high-quality isoforms and ASIs.
